# Potassium in dental calculus as an indicator of diabetes

**DOI:** 10.1186/s12903-025-07442-9

**Published:** 2026-01-10

**Authors:** Z. Szepesi, R. Kállai, A. Csík, I. Varga, K. Tőkési

**Affiliations:** 1 Department of Periodontology, Faculty of Dentistry – University of Debrecen, Debrecen, EU Hungary; 2 HUN-REN Institute for Nuclear Research, (ATOMKI), Debrecen, EU Hungary

**Keywords:** Diabetes mellitus, Dental calculus, Potassium, SEM-EDX, Biomarker

## Abstract

**Background:**

Diabetes Mellitus (DM) is a chronic metabolic disorder that has been linked to an increased risk of periodontal disease. Among the oral manifestations associated with DM, periodontal disease has been the most extensively studied. Dental calculus, which forms when dental plaque mineralizes over time, is known to trap biological substances and may preserve biochemical markers indicative of systemic health. Therefore, it could provide valuable information about patients’ overall health. In this study, we analyzed the elemental composition of dental calculus in diabetic and non-diabetic (control) patients using scanning electron microscopy (SEM) combined with energy dispersive X-ray spectroscopy (EDX), with a specific focus on potassium (K) content.

**Patients and methods:**

We analyzed 57 dental calculus samples, including 17 from individuals diagnosed with type 2 diabetes and 40 from non-diabetic controls. The elemental composition was assessed using scanning electron microscopy paired with energy-dispersive X-ray spectroscopy (SEM-EDX), targeting standardized 100 × 100 μm regions. Potassium content, along with other elemental data, was reported in both mass and atomic percentages.

**Results:**

The results showed elevated potassium levels in diabetic patients compared to controls, with the difference reaching the treshold of statistical significance (0.484 ± 0.710 wt.% versus 0.186 ± 0.320 wt.%). A similar trend was observed in atomic percentage values (0.249 ± 0.387 versus 0.092 ± 0.174; *p* = 0.0555). Notably, potassium was present in 100% of the diabetic samples. Carbon and oxygen were the predominant elements in both groups, with the diabetic group exhibiting a slight increase in oxygen and lower levels of calcium and phosphorus.

**Conclusion:**

These findings suggest that potassium concentration in dental calculus could serve as a potential biomarker for diabetes. They also highlight the feasibility of incorporating dental calculus analysis after routine dental treatments— which involve the removal of calculus—for early diabetes detection. This approach provides a cost-effective and painless alternative to traditional diagnostic methods. However, we note that while the data are still preliminary, the results indicate a potential biochemical link that warrants further exploration in larger, well-controlled studies.

## Introduction

Diabetes mellitus is a chronic metabolic disorder characterized by impaired glucose metabolism, systemic inflammation, and increased risk of oral diseases. Periodontal disease, in particular, occurs more frequently and more severely in diabetic patients [1].

While dental plaque is the primary etiological factor in periodontal pathology, dental calculus *–* its mineralized form *–* serves as a secondary contributing factor by facilitating persistent plaque retention and limiting effective oral hygiene [2, 3]. Beyond microbial deposits, calculus can retain salivary and blood-derived biochemical components, including electrolytes such as calcium, phosphorus, and potassium [4].

Altered salivary electrolyte profiles have been reported in diabetic patients, though findings remain inconsistent. Variations in the concentrations of potassium, calcium, phosphate, and sodium have been noted across studies. Elevated potassium levels have been observed frequently, raising interest in their potential diagnostic relevance [5–7].

Recent microbiological research has identified potassium as a signaling ion within the oral microbiome. Potassium ion flux can modulate microbial gene expression, promote virulence factors, and influence the host immune response *–* e.g., stimulating cytokines such as IL-6 and TNF-*α* [8]. Whether these shifts are reflected in dental calculus over time remains unclear.

The aim of our recent studies is to investigate potassium concentrations in dental calculus from diabetic and non-diabetic patients using SEM-EDX. While previous studies have primarily focused on salivary potassium, dental calculus offers a fundamentally different analytical substrate. Unlike saliva, which reflects transient physiological states, dental calculus may reflect biochemical changes over longer periods [2], offering a cumulative record of both oral and systemic conditions. We demonstrate the correlation of potassium concentration in samples collected from diabetic and non-diabetic patients.

## Materials and methods

### Study design and participants

Our study analyzed 57 dental calculus samples, 17 from patients diagnosed with type 2 diabetes and 40 from non-diabetic patients. Due to the exploratory nature of the study and the limited availability of qualified calculus samples, the sample size was determined by the number of eligible specimens collected during the study period. Approval for the research was granted by the Regional and Institutional Research Ethics Committee of the Clinical Center of the University of Debrecen on June 23, 2016, under the protocol DEOEC RKEB/IKEB Prot. No. 4602 − 2016. Reporting of all experimental procedures adhered to the recommendations of the International Council for Harmonisation of Technical Requirements for Pharmaceuticals for Human Use (ICH) and good clinical practice (GCP) guidelines. All participants provided written informed consent.

Although strict one-to-one matching was not applied, attempts were made to balance the age and gender distribution across diabetic and control groups.

### Demographics

- Diabetic patients (*n* = 17): 8 women, 9 men; mean age: 49 years.
- Non-diabetic controls (*n* = 40): 19 women, 21 men; mean age: 47.5 years.
- Smoking: 16 individuals (all from the control group).

To assess potential influences on periodontal health, oral hygiene behavior was documented.

(see Table 1.).

**Table 1:** Comparison of periodontal health, oral hygiene for diabetic and non-diabetic patients

Oral hygiene behavior	Diabetic patients	Controls
Symptom-driven dental visits	47%	45%
Never had scaling	35%	27.5%
Regular use of interdental cleaning tools (floss, interdental brush)	29%	45%

Although comprehensive periodontal assessments were not conducted, both groups demonstrated generally poor oral hygiene, with slightly poorer indicators in the diabetic group. These variations were documented to allow contextual interpretation of results and are acknowledged as a potential limitation of the study.

### Sample collection and handling

Dental calculus samples were collected during routine scaling procedures using sterile Gracey curettes following standard procedure, as described by Lindhe et al. [9]. After isolation of the area with cotton rolls, calculus deposits were gently removed without disrupting adjacent soft tissue. Each sample was immediately placed in a sterile, labeled Eppendorf tube and stored dry at room temperature. Samples were transported to the analytical laboratory within 24h of collection, under dry conditions at room temperature, to prevent contamination and preserve sample integrity.

### SEM-EDX analysis

Dental calculus samples were collected at the University of Debrecen, Faculty of Dentistry (Debrecen, Hungary), and subsequently analyzed at the HUN-REN Institute for Nuclear Research and the Faculty of Dentistry. To investigate surface morphology and elemental composition, Scanning Electron Microscopy (SEM) coupled with Energy-Dispersive X-ray Spectroscopy (EDX) was employed.

Prior to analysis, samples were cleaned, air-dried, and mounted on carbon-based double-sided adhesive tape. To minimize changing effects during imaging of the non-conductive calculus surfaces, all specimens were sputter-coated with a ~ 15 nm thick layer of gold (Au) using a Bio-Rad E5000C sputter coater.

SEM analysis was performed using a Hitachi S-4300 field emission scanning electron microscope operated at an accelerating voltage of 15 kV. Initial low-magnification imaging provided an overview of the surface topography, followed by higher magnification imaging (x700 and x2500) to examine surface microstructure.

For elemental analysis, the energy dispersive X-ray (EDX) method was employed. The elemental composition was measured as accurately as possible on a randomly selected 100 × 100 μm flat surface to minimize the influence of surface irregularities on compositional measurements. Additionally, the samples were positioned appropriately and rotated or tilted as needed. During the composition analysis, the detection threshold was 0.1 at.%.

### Statistical analysis

Mean potassium concentrations, both mass% (wt.%) and atomic% (at.%), were calculated for diabetic patients and the control group. The primary outcome was potassium content expressed as mass percentage (K wt.%) and atomic percentage (K at.%). Group comparisons were performed using one-tailed Welch’s t-test, based on the hypothesis that diabetic patients would exhibit higher potassium levels.

Diabetic patients showed much higher average potassium concentration than the control group as can be seen in Table 2.

**Table 2:** Average potassium concentration in the investigated samples for diabetic and non-diabetic patients

	Diabetic patients	Controls	*p*-value
K, wt.%	0.484 ± 0.710	0.186 ± 0.320	0.0500
K, at.%	0.249 ± 0.387	0.092 ± 0.174	0.0555

While statistical significance was marginal for potassium mass percentage, both measures indicate a trend toward elevated potassium in the diabetic group. A *p*-value below 0.05 was considered statistically significant.

### Statistical summary

To further interpret the group differences, Cohen’s d was calculated as a measure of effect size. For potassium Cohen’s d values were 0.66 (wt.%) and 0.64 (at.%), indicating moderate effect sizes. These results suggest that the differences in potassium levels are statistically suggestive and potentially clinically relevant, supporting the potential utility of potassium as a biomarker in dental calculus for diabetic patients.

## Results

**Fig. 1:**
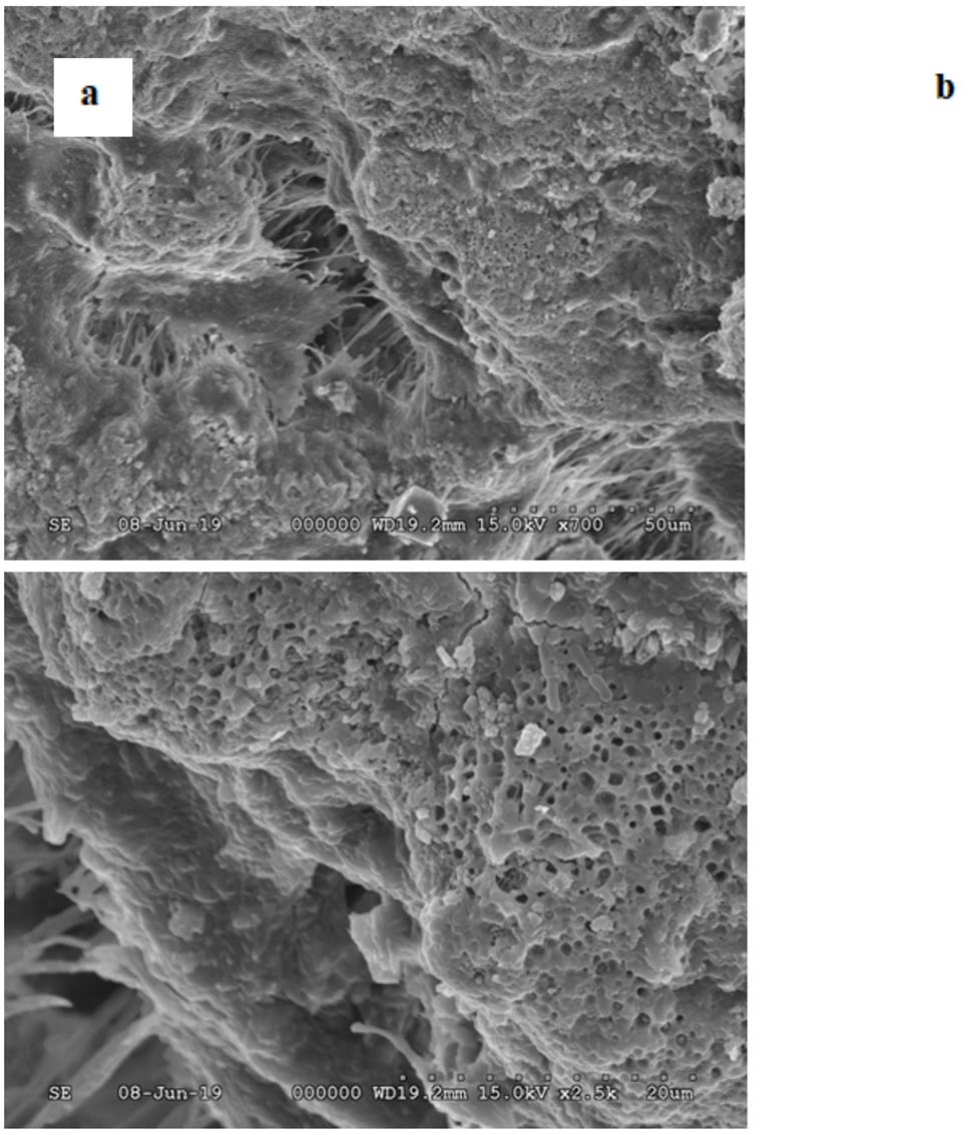
SEM micrographs of dental calculus. **a** Surface morphology at x700 magnification showing compact and rough regions. **b** Higher magnification (x2500) reveals porous areas with 0.5–1 μm holes

SEM imaging revealed heterogeneous surface morphology in dental calculus, characterized by irregular, coarse structures interspersed with smoother regions. Some areas exhibited compact and homogeneous features, while others showed pronounced porosity with voids ranging from 0.5 to 1 μm in diameter. No obvious morphological difference was observed between samples of diabetic patients and non-diabetic controls (Fig. 1a, b).

The EDX analysis identified the presence of key elements, including carbon (C), oxygen (O), calcium (Ca), phosphorus (P), potassium (K), magnesium (Mg), and aluminum (Al). A prominent carbon signal was detected, likely arising from both residual organic matter and the sample coating procedure. This signal was attributed to the gold sputtered coating applied during sample preparation. Potassium was detected in all of the samples from diabetic patients.

**Fig. 2:**
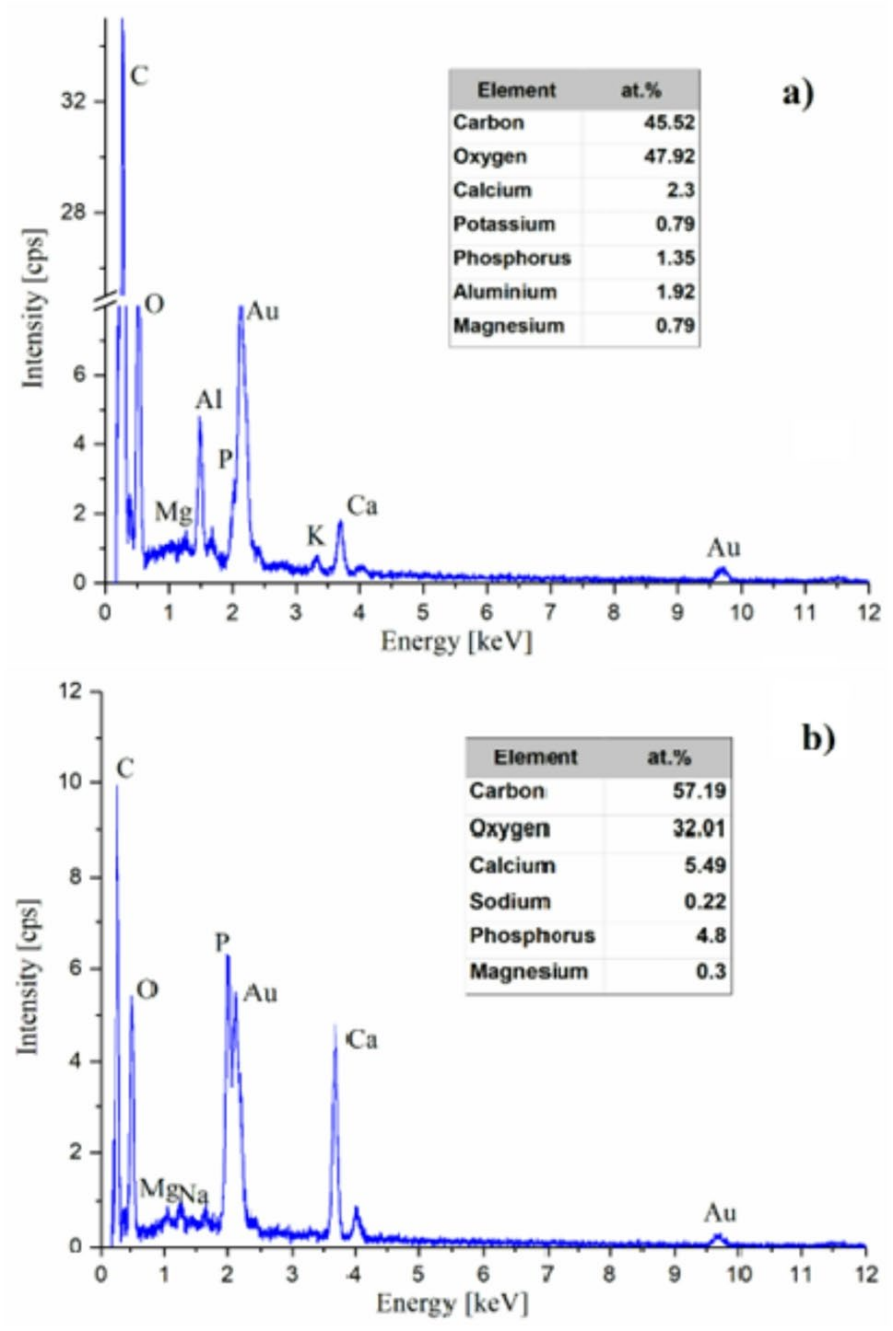
Representative EDX spectra measured on 100×100 μm areas of the sample surface. **a** Sample from a diabetic patient showing distinct potassium peaks. **b **Sample from a non-diabetic patient with no detectable potassium signal

Figure 2 displays representative EDX spectra: one from a diabetic patient, showing a visible potassium peak (Figure 2a), and one from the control group (Figure 2b), where potassium was not detected.

## Discussion

In this study, we observed a trend toward higher potassium levels in dental calculus from diabetic patients compared to non-diabetic controls. These results align with previous observations of altered salivary potassium in diabetic patients [6, 7] and extend this association to dental calculus, suggesting potential for retrospective biomarker analysis.

Prior studies have reported increased salivary potassium levels in diabetic patients [5], possibly due to acinar cell dysfunction or altered glandular regulation [10]. Once incorporated into forming calculus, potassium may provide a cumulative record of these systemic changes. In this context, our findings support the hypothesis that dental calculus can reflect chronic metabolic influences.

While our findings indicate a trend of elevated potassium in the dental calculus of diabetic patients, it is important to recognize that the underlying incorporation of electrolytes into maturing plaque remains incompletely understood. Although we cannot directly trace the origin of potassium deposits, it is reasonable to assume that elevated salivary potassium contributes to the accumulation of this element during plaque mineralization.

Recent studies highlight the role of potassium as a key environmental signal in the development and progression of periodontal disease. Elevated extracellular potassium levels, similar to those observed in deep periodontal pockets, can trigger functional changes in subgingival biofilms. These changes include the upregulation of microbial genes involved in proteolysis, motility, and iron metabolism, all of which are closely linked to tissue destruction. At the same time, gingival epithelial cells exposed to high potassium concentrations exhibit increased expression of pro-inflammatory cytokines such as TNF-*α*, reduced production of protective peptides like human *β*-defensin-3, and activation of pro-inflammatory pathways such as the NLRP3 inflammasome [8, 11]. Together, these responses contribute to a pro-inflammatory, dysbiotic microenvironment and may amplify both microbial and host-driven mechanisms of periodontal tissue breakdown. This positions potassium not only as a potential marker of disease but also as an active contributor to its pathogenesis.

Beyond serving as a nidus for plaque retention, the mineral content of dental calculus may also contribute directly to periodontal inflammation. Although potassium is not a major structural component due to its high solubility and limited capacity to form stable precipitates like calcium or phosphate, it may still influence the surrounding microenvironment. Potassium ions (K^+^) loosely associated with the mineralized matrix could leach into the gingival crevice, particularly in inflamed or mechanically disturbed sites [12]. Once released, these ions may contribute to a local milieu that favors dysbiosis and tissue breakdown. Potassium embedded in calculus may act as a persistent inflammatory signal, even if not fully immobilized, contributing to the progression of periodontal disease.

Due to hyperinflammatory responses and altered immune regulation commonly observed in diabetic patients [13], these potassium-driven effects may be even more pronounced. The potential activation of potassium-sensitive inflammatory pathways may therefore further exacerbate periodontal tissue destruction in this population.

We also observed lower calcium and phosphorus levels in diabetic samples, which may reflect shifts in salivary pH or mineral availability. However, definitive conclusions are limited by the study’s cross-sectional design.

It is also important to acknowledge several limitations. Our sample size was modest and lacked control for dietary potassium intake, medications, and kidney function, which also affect potassium levels. In addition, the EDX technique measures elemental composition only on the surface layer, and future volumetric or longitudinal analyses would strengthen these observations.

From a clinical perspective, these findings contribute to our understanding of systemic-oral interactions in diabetes. Although calculus is not practical for routine chair-side diagnostics, its elemental profile could provide valuable information for research exploring the systemic-oral health connection in diabetes [2].

## Conclusion

The elemental analysis of dental calculus in diabetic and non-diabetic control patients was conducted using Scanning Electron Microscopy. Our findings indicate that diabetes is associated with elevated potassium concentrations in dental calculus. In samples from non-diabetic patients, potassium levels were either undetectable or very low. Conversely, higher potassium levels were a clear indication of diabetes. Our results suggest that dental calculus can serve as an effective indicator of diabetes, even in the early stages of the disease. This discovery paves the way for cost-efficient and painless diabetes screening techniques that could be integrated into routine dental visits. To the best of our knowledge, this is the first study to detect diabetes through dental calculus analysis. Our findings support the idea that dental calculus may serve as a biomarker for systemic conditions such as diabetes mellitus. Given the established links between oral health, salivary composition, and systemic disorders—including renal, cardiovascular, and respiratory diseases—further investigation of calculus-based biomarkers is warranted. Further studies involving larger, well-characterized populations are necessary to validate these findings, explore their clinical applications, and support the development of personalized preventive strategies for individuals at elevated systemic risk.

## Data Availability

The datasets generated and/or analyzed during the current study are available from the corresponding author on reasonable request.

## Abbreviations

IL-6: Interleukin-6
TNF-α: Tumor necrosis factor alpha
hBD-3: Human *β*-defensin-3
GCP: Good clinical practice
ICH: International Council for Harmonisation of Technical Requirements for Pharmaceutical for Human Use
SEM: Scanning Electron Microscopy
EDX: Energy Dispersive X-ray

## References

[1] Chan HH, Rahim ZHA, Jessie K, Hashim OH, Taiyeb Ali TB. Salivary proteins associated with periodontitis in patients with type 2 diabetes mellitus. Int J Mol Sci. 2012;13(4):4642*–*54. 10.3390/ijms13044642. 10.3390/ijms13044642 PMC3344237 22606001

[2] Forshaw R. Dental calculus *–* oral health, forensic studies and archaeology: a review. Br Dent J. 2022;233(11):961*–*7. 10.1038/s41415-022-5266-7. 10.1038/s41415-022-5266-7 PMC9734501 36494546

[3] Rahim A, Hassan S, Ullah N, Khan U, Iqbal M, Ahmed N. Association and comparison of periodontal and oral hygiene status with serum HbA1c levels: a cross-sectional study. BMC Oral Health. 2023;23:442. 10.1186/s12903-023-03042-7. 10.1186/s12903-023-03042-7 PMC10316548 37394484

[4] Saini R, Saini S, Sharma S. Dental calculus: a strategic review. Int J Dent Health Sci. 2014;1(5):788*–*95.

[5] Shirzaiy M, Heidari F, Dalirsani Z, Dehghan J. Estimation of salivary sodium, potassium, calcium, phosphorus and urea in type II diabetic patients. Diabetes Metab Syndr Clin Res Rev. 2015;9:332*–*6. 10.1016/j.dsx.2013.02.025. 10.1016/j.dsx.2013.02.025 25470630

[6] Mata AD, Marques D, Rocha S, Francisco H, Santos C, Mesquita MF, et al. Effects of diabetes mellitus on salivary secretion and its composition in humans. Mol Cell Biochem. 2004;261(1*–*2):137*–*42. 10.1023/b:mcbi.0000028748.40917.6f. 10.1023/b:mcbi.0000028748.40917.6f 15362496

[7] Kallapur B, Ramalingam K, Bastian A, Mujib A, Sarkar A, Sethuraman S. Quantitative estimation of sodium, potassium, and total protein in saliva of diabetic smokers and nonsmokers: a novel study. J Nat Sci Biol Med. 2013;4(2):341*–*5. 10.4103/0976-9668.117006. 10.4103/0976-9668.117006 PMC3783777 24082729

[8] Yost S, Duran Pinedo AE, Krishnan K, Frias-Lopez J. Potassium is a key signal in host*–*microbiome dysbiosis in periodontitis. PLoS Pathog. 2017;13(6):e1006457. 10.1371/journal.ppat.1006457 PMC5493431 28632755

[9] Lindhe J, Lang NP, Karring T. Clinical Periodontology and Implant Dentistry. 6th ed.Oxford: Wiley-Blackwell; 2015.

[10] Cards C, Mosquera Lloreda N, Salom L, Ferraris MEG, Peydró A, Structural and functional salivary disorders in type 2 diabetic patients. Med Oral Patol Oral Cir Bucal. 2006;11(4):309*–*14. 16816810

[11] Montenegro JL, Amorim RM, da Silva MJ, Cunha LD, Zamboni DS, Zorzetto-Fernandes AL, et al. Dental calculus stimulates interleukin-1β secretion by activating NLRP3 inflammasome in human and mouse phagocytes. PLoS One. 2016;11(9):e0162865. 10.1371/journal.pone.0162865. 10.1371/journal.pone.0162865 PMC5025015 27632566

[12] White DJ. Dental calculus: recent insights into occurrence, formation, prevention, removal and oral health effects of supragingival and subgingival deposits. Eur J Oral Sci. 1997;105(5):508–22. 10.1111/j.1600-0722.1997.tb00238.x. 10.1111/j.1600-0722.1997.tb00238.x 9395117

[13] de Candia P, Prattichizzo F, Garavelli S, Matarese G, della Valle V, Santoro C, et al. Type 2 diabetes: how much of an autoimmune disease? Front Endocrinol (Lausanne). 2019;10:451. 10.3389/fendo.2019.00451. 10.3389/fendo.2019.00451 PMC6620611 31333589

